# Patterned activation of action potential patterns during offline states in the neocortex: replay and non-replay

**DOI:** 10.1098/rstb.2019.0233

**Published:** 2020-04-06

**Authors:** Tang-Yu Liu, Brendon O. Watson

**Affiliations:** Department of Psychiatry, University of Michigan, Biomedical Science Research Building, 109 Zina Pitcher Place, Ann Arbor, MI 48109, USA

**Keywords:** cortex, sleep, replay, memory, homeostasis, plasticity

## Abstract

Action potential generation (spiking) in the neocortex is organized into repeating non-random patterns during both awake experiential states and non-engaged states ranging from inattention to sleep to anaesthesia—and even occur in slice preparations. Repeating patterns in a given population of neurons between states may imply a common means by which cortical networks can be engaged despite brain state changes, but super-imposed on this common firing is a variability that is both specific to ongoing inputs and can be re-shaped by experience. This similarity with specifically induced variance may allow for a range of processes including perception, memory consolidation and network homeostasis. Here, we review how patterned activity in neocortical populations has been studied and what it may imply for a cortex that must be both static and plastic.

This article is part of the Theo Murphy meeting issue ‘Memory reactivation: replaying events past, present and future’.

## 1. Introduction

Action potential generation is thought by neuroscientists to underlie perception and decision making during active behaviour (‘online’ state), but it is also thought to underlie many other neural processes including memory consolidation and homeostasis during ‘offline’ brain states. ‘Offline’ states can be defined as those in which active interaction with the world is reduced and is usually accompanied by behavioural inactivity or sleep. Notably, the brain is not ‘off’ during these inactive states as it generates action potentials, often in patterned sequences that may correspond to specific tasks and/or may reshape neural circuits via synaptic learning rules. This reshaping of neural circuits may then define the brain activity, memory, larger cognition and actions of the organism into the future.

Patterned offline generation of spikes can be termed ‘reactivation’ or ‘replay’ when the sequences and patterns observed recapitulate prior spiking activity. Replay and reactivation have been studied most extensively in the hippocampus but may have important roles in the neocortex. In this review, we will explore the role of patterned activations in neocortical circuits.

## 2. Definitions

Here, we will consider sequences, reactivations and replay as three separate types of neural events. *Replay* can be considered to be a full recapitulation during an offline state of spiking patterns that originally were driven by experience during an ‘online’ behaving state. Replay includes repeated relative timing of spiking activity between cells from online to offline states. This is essentially the most complete repetition of activity considered here given that it involves both which cells fire and when they fire. Of note, in some cases, only relative timing (or rank sequence of cells) may remain intact while absolute timing changes, meaning there is a stretching or squeezing of the overall duration of the sequence.

*Sequences* are in some ways similar events to replay in that they include repeated relative spike timing between neurons, but they do not necessarily reflect previous behaviour-linked firing patterns.

Finally, *reactivation* here will be considered to be spiking by the same neurons with approximately the same relative firing rates per cell during offline states as were observed during online states—but without specified temporal sequence patterns. Often non-report of sequence repetition in reactivation experiments may be owing to lack of sequence examination by researchers.

Therefore, spike sequences (timing but not based on prior events) and reactivation (recapitulation of prior rates but not necessarily timing) can be considered to be sub-elements of full replay.

Finally, replay of waking sequences during offline states may or may not be influenced by recent experience. In fact, much replay, especially in the cortex occurs in a manner that does not appear to be influenced by recent experience but rather recapitulates some default network state and therefore may not relate to a memory consolidation role.

The rodent hippocampus is the neural structure in which replay is best studied and work there serves as an important guide to cortical spike sequence researchers. As a result, we will briefly review that literature before proceeding to neocortical experiments.

## 3. Replay in the hippocampus

The hippocampal sharp wave-ripple event (SWR) is the best described neurophysiologic correlate of replay [1]. During these offline events, neurons tend to fire with the same relative timing to each other as they did during waking behaviour [2–8]. The best-known SWR replay experiments in the hippocampus focus on location-encoding place cells thereby allowing researchers to specifically map replay events onto behaviour-related maze running events. For example, on a maze task, an animal may run repeatedly from place A to B to C, encoded by place cells A′, B′ and C′, respectively. The running behaviour may take place over the course of multiple seconds driving place cells A′, B′ and C′ to fire in a sequence over those seconds owing to external drive from behaviour. During SWR events, as characterized by their local field potential (LFP) oscillations of approximately 150 Hz, neurons tend to fire at elevated rates and furthermore many groups have reported increased replay of the A′, B′, C′ temporal sequence compared to both prior to the ABC running experience and compared to reshuffled spiking data. This replay is often at reduced timescales (say 5–15 fold reduced, i.e. from seconds down to less than 100 ms) and with reduced numbers of total action potentials [2,9,10]. These compressed sequences may occur during SWRs that happen either during offline waking states such as during eating or drinking [11,12] or during non-rapid eye movement (nonREM) sleep when SWRs are most common [2,6,9,13].

Importantly, the awake behaving experience of an animal can shape the subsequent replay activity during SWRs in the hippocampus [3,6,8], despite the fact that non-random firing sequences can be found in hippocampal populations prior to experimenter-introduced experience—a phenomenon known as ‘preplay’ [3,5,14]. The spiking sequences observed during the novel and salient experiences, such as maze running to obtain water in a thirsty rat, for example, tend to be the same sequences that become increasingly represented in subsequent offline SWRs [6]. This ability of experience to reshape offline activity in hippocampal networks has led to the idea that SWR-based replay may represent a critical mechanism for Hebbian memory consolidation.

Specifically, this conception of consolidation states that SWR spike sequences can be influenced by online activity so that the SWR can replay spiking sequences offline to sculpt the synaptic structures of networks [1,10,15,16]. The capacity for this repeated spiking to reshape synaptic structures is undergirded by spike-timing-dependent plasticity (STDP) [17], a phenomenon described mostly *in vitro* wherein neurons that fire within milliseconds or tens of milliseconds of each other on a reliable basis will demonstrate plasticity at synapses between them. Specifically, if a pre-synaptic neuron fires approximately 1–20 ms before a postsynaptic neuron, the synapse between them will strengthen or potentiate. On the other hand, if the neurons fire in the opposite sequence with the postsynaptic neuron firing first, the synapse will weaken. For this to occur, neurons must fire within milliseconds, and therefore the compressed timescale of sequential firing during SWRs may enable plasticity to occur between replaying neurons that might fire seconds apart during waking experience. Evidence for these SWR events causally playing a role in memory consolidation comes from experiments in which SWRs were interrupted and memory was reduced [18,19] or prolonged and memory improved [20].

Might similar events occur in the neocortex?

## 4. Multi-neuronal sequences in neocortical slices

Some of the earliest evidence of multi-neuronal sequences in the neocortex came from calcium imaging experiments in neocortical slices. In these experiments, hundreds of neurons were continuously monitored with non-genetically encoded calcium indicator dyes and repeating patterns of activation were observed. An initial study [21] based simply on long-term time-lapse imaging of unstimulated neocortical slices observed large groups of neurons spontaneously activating within approximately 1 s, simultaneously with intracellular depolarization events. These 500–1000 ms duration intracellular depolarizations were reminiscent of nonREM sleep UP states, which can be described similarly and occur approximately once per second during most of the sleep [22]. Subsequent work at higher temporal resolution, still in slices, showed that these multi-neuronal activations during UP state-like depolarizations involved repeating multicellular firing sequences [23]. Because this work was performed in primary somatosensory cortex with intact projection axons from the somatosensory thalamus, these spontaneous firing patterns were compared against those induced by stimulation of the thalamus and were found to be identical regardless of how they were initiated. Additional experiments to attempt to interrupt these neocortical sequences by delivering thalamic inputs during the sequence were unsuccessful with patterns largely unperturbed regardless of whether novel inputs were given [24]—seemingly owing to a high conductance in neurons during the UP state-like depolarizations [25]. Work in visual cortex also implied repeating cortical sequences there as well [26,27].

These works collectively suggest that the sequences observed in these neocortical slices were based on local network properties, because neurons fired in the same sequence regardless of whether they were triggered or initiated spontaneously—and were difficult or impossible to interrupt. Attempts to alter patterns of activity by ablating up to dozens of the local neurons involved in the typical repeating patterns were additionally unsuccessful (B.O. Watson, R. Yuste 2007, unpublished observations), again implying a strong and collective network drive to these patterns in a local sliced circuit.

## 5. *In vivo* sequences during offline states

While sequences have been studied during many online task-engaged states [28,29], we focus here on sequences generated during offline states. These are in some ways analogous to the brain slices discussed above, though of course, they occur in the milieu of the full brain network, neuromodulators, LFPs and other elements.

**Figure 1.**
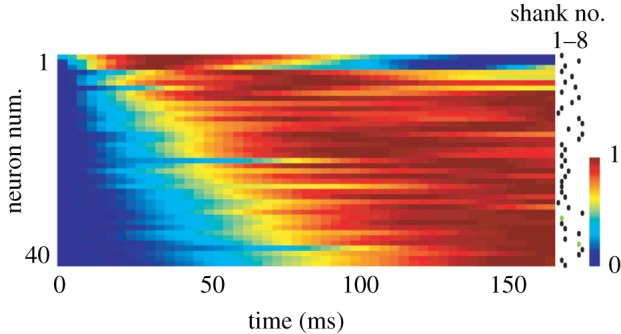
Neuronal firing sequence at UP state onset. Somatosensory cortical neuronal populations in rats were recorded using 8-shank silicon probe electrodes during nonREM. The normalized peri-event time histogram (PETH) is shown aligned with the UP state onset time with each row a neuron and colour denoting averaged spike rate of that neuron over all UP states. The neurons are ordered by the latency, or the centre of mass of the PETH. The dots on the right showed in which shank the neurons were recorded. Adapted from [32]. (Online version in colour.)

UP states in live animals occur prominently during nonREM sleep (though also during certain waking states and anaesthesia) and represent one half of a semi-regular fluctuation between a depolarized UP state with population spiking and a hyperpolarized generally non-spiking DOWN state occurring synchronously across multiple neurons in a local neocortical region [22,30]. This UP/DOWN fluctuation seen in membrane potential recordings corresponds exactly with the extracellularly measured slow oscillation with delta waves in the LFP corresponding to DOWN states [30] and multi-neuronal spiking occurring among extracellular units simultaneously with UP states [31]. Thus, the multi-neuronal firing activity during UP state depolarizations in these sleep oscillations *in vivo* may relate to the depolarized multi-neuronal firing *in vitro*. To directly examine this Luczak *et al.* [32] used silicon probe electrode arrays in cortex to record during both anaesthetized and natural sleep slow oscillations and found indeed that multi-neuronal patterns repeated from one activation to the next (figure 1). Specifically, they found that at the transition from hyperpolarized DOWN states to depolarized UP states, or the ‘DOWN to UP transition’, certain neurons tend to consistently fire earlier and others later.

Subsequently, these authors examined the relationship between these spontaneous sleep activations and sensory-evoked spiking sequences [33,34]. They found that stimulated firing events in auditory cortex shared firing sequences with those found during natural sleep [33]. These sequence events are present without clear experience and remain throughout the experiment and so are not necessarily driven by the recent experience and may not represent the consolidation of previous memories as in SWR-based replay.

## 6. The repeated sequence as a default neocortical network state

The repetition of sensory-evoked patterns during offline sleep may be owing to the fact that sensory stimulation induces UP state-like membrane dynamics [31,35] and therefore similar mechanisms may be engaged. Of note, UP state dynamics with repeating sequences are seen in both slices [24] and in anaesthetized animals [32] and may, therefore, represent an innately activatable network state. Hence anaesthetized, nonREM-based and sensory-evoked UP states all show overlapping sequence activations and all of these cases share the feature of a relatively prolonged silence (greater than 70 ms) prior to activation. Therefore, the neocortical network may have features pre-disposing it to similar network activation patterns upon delivery of inputs after a silence regardless of the context of that silence.

The capacity of these neocortical networks to repeat firing when activated via different means fits nicely with a perspective that spontaneous activity should recapitulate the sensory-receptive state, and yet it also suggests that there may be some inflexibility to the cortical network. It is not initially clear how such a network can be influenced by recent activity, for example. This apparent inflexibility may be of some intellectual concern to those looking to the cortical network to be adaptive. These sequences are clearly present, but on the other hand, they are not adhered to closely during each individual UP state event—rather they become statistically evident after averaging together many events, often hundreds [33,36,37]. This means that there may be a generalized backbone sequence that tends, on average to be adhered to, but that on a single event basis much variation may occur, and that variation may carry important information. In fact, we define our concept of ‘backbone sequences’ as being those sequences that can be measured by taking the average spike timing of all UP state events, despite the variance of individual events around this average.

Overall then, the sequences seen in the cortex may be the result of an in-built network state that becomes activated whenever the network is activated from a long-term inactive state—be that by thalamic input in slices, by sensory inputs *in vivo*, during anaesthesia or during nonREM sleep. The fact that this backbone sequence is not rigorously followed but is rather loosely adhered to, may allow the capacity for additional information to be transmitted by the specific firing of individual sequence event. In fact, the ‘backbone’ sequence may at least in part represent a ‘carrier wave’ or means of basic activation and propagation of the network, while the variable elements of the sequence may represent the information. This is similar to elements of the idea of ‘packets’ introduced in the literature [38], but with some differing implications as we explore this further below.

Importantly, the above-described experiments do not demonstrate firing influenced by actively task-engaged behaviour. We, therefore, discuss neocortical reactivations next, where such linkage is more clearly present.

## 7. Reactivation in the neocortex

Offline reactivation of patterned activity initially established during the recent waking experience can signify plasticity in two ways. First, the capacity to repeat a recently imposed spiking regime in itself shows an initial ability to learn and reshape network activity. Second, the purpose of such repetition might be to subsequently consolidate memories via STDP-based synaptic restructuring [39]. Therefore, reactivation of learned spike sequences is both a sign of extant plasticity and also may be a sign of a process to reinforce that plasticity. It has long been hypothesized that consolidation of memories might happen during sleep [39] given evidence of memory refinement after sleep [40].

First, a beautiful study explores the bounds of spike rate-based reactivation (though not replay) in detail in the auditory cortex [33]. These experiments combine spontaneous activity, evoked responses to simple auditory tones and evoked responses to more complex natural auditory stimuli. First, natural sounds, tones and spontaneous events were initially shown to evoke events with similar spiking—including, in fact, timing-based sequences. Later the authors used time bin-based firing rate vectors of their recorded populations to explore the multi-neuronal firing rate space occupied by tones, natural stimuli and spontaneous activity (rate of each neuron represented by a dimension in multi-dimensional space). They found that while the spontaneous firing rate vectors only occupied a sub-space of the firing rate vectors theoretically possible, that set of spontaneous vectors actually contained in it all of the natural and tone response rate vectors. This means that the spontaneously active rate vectors showed a mix of similarity and variability, and that within that set of variable responses are contained the sensory-inducible population responses.

Evidence of recent activity sculpting reactivation comes from a study of the medial prefrontal cortex (mPFC) neurons recorded during, before and after a choice-based navigation task [41]. Here, neuronal spike rates in 100 ms bins observed during wake were reactivated during post-task sleep more than they were during pre-task sleep. Specifically, groups of neurons tended to re-activate with the same relative spike rates seen in the task during post-activity sleep in a manner they did not during pre-activity sleep. Similar findings were then replicated across a variety of tasks and neocortical regions [42–44]. These studies were based on a method wherein time is binned (say 50 ms bin width, though this is arbitrary) and neurons that tend to fire together in coherent assemblies (as signified by consistent between-neuron correlations of relative spike rates) can be recognized as co-activating assemblies using principle components analysis [36]. While this method does not yield information about the sequential replay, it has proven robust and has become more widely used given its ease of implementation. It has also been refined to include independent components so that it can better recognize non-orthogonal assemblies/principle components [45].

What of full sequences in the neocortex that replay recent experience?

## 8. Replay of sequences in the neocortex

Replay of wake-experienced sequences of neuronal firing patterns during offline states represents essentially the best-measurable recapitulation of firing activity. There is good evidence that it occurs not just in the hippocampus but in the neocortex as well: in both primary sensory cortices and in association cortices such as the mPFC.

In prefrontal cortical recordings, Euston *et al.* [46] showed that neurons with place-related firing patterns showed task-imposed structured spiking during repeated behaviours. These sequences were then repeated during subsequent slow-wave sleep—and more than in previous sleep. The replayed sequences were actually replayed at approximately six- to sevenfold increased speed relative to their original waking sequence speed, reflecting what appeared to be fast-forward replay in concordance with similar phenomena in the hippocampus [2,10].

In primary somatosensory cortex, innate spike sequences during slow-wave UP states were able to be re-sculpted based on novel somatosensory input [47]. However, these happened not across wake and sleep states, but rather in anaesthetized states with and without amphetamine to simulate wake and sleep, respectively. The authors first recorded a pre-sleep-like non-amphetamine anesthetized state with baseline sequences displayed. In the same session, they then recorded an amphetamine-based wake-like state during which somatosensory stimuli were given that induced different firing sequences which differed somewhat from that in the previous sleep-like state. Finally, they recorded a post-sleep-like state without amphetamine wherein spontaneously replayed had come to resemble the stimulated sequences. This did not occur if amphetamine was not given during the stimulus, which showed the state-dependent sculptability.

**Figure 2.**
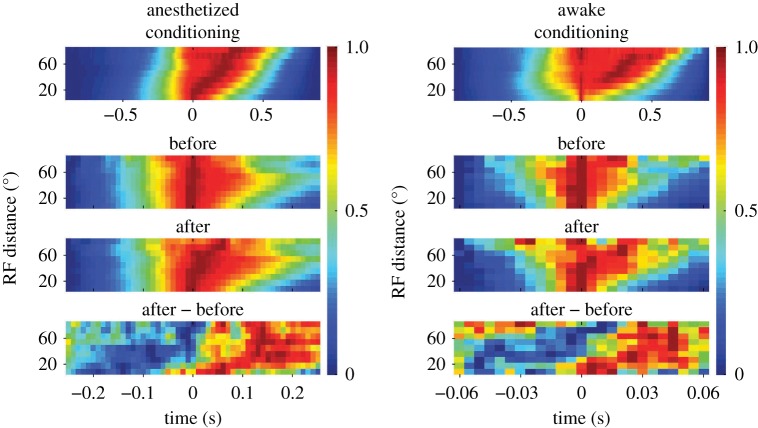
Conditioning-induced increase of similarity in sequential spiking for anaesthetized (left) and awake (right) rats. V1 neurons are evoked by repeated stimulation with a moving visual stimulus. After conditioning, a brief flash at the beginning of the moving trajectory could evoke a sequential firing pattern that is similar to the previous evoked activation order. The evoked responses to the flash only at the beginning are compared between before and after conditioning. The top three rows are the averaged pairwise cross-correlation of a certain unit against all other recorded units, and the unit is ordered by the distance between its receptive field (RF) centre and the beginning spot. Normalized cross-correlation amplitude is indicated in colour. The bottom row represents the difference of the cross-correlation before and after conditioning. Note the shorter timescale in re-sculpted activities compared with the conditioning. Adapted from [29]. (Online version in colour.)

**Figure 3.**
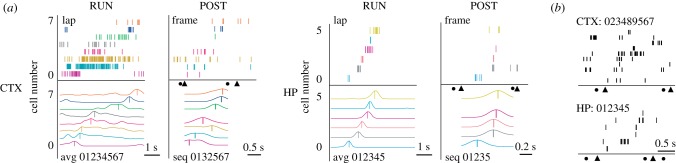
Sequence replay in the visual cortex (CTX) and the hippocampus (HP). (*a*) Firing sequence rasterplot during a maze run is replayed during post run sleep (POST) in both CTX (left) and HP (right). The curves at the bottom represent smoothed firing rates based on rasters above, with firing peaks denoted by vertical lines. The order of the peak timings is compared between RUN and POST. Triangles and circles represent start and end times of analysed spiking ‘frames’. Note compressed time base of POST replay versus initial RUN events. (*b*) CTX and HP replays overlap in time and can be simultaneous. Adapted from [48]. (Online version in colour.)

In visual cortex, extended visual experiences were able to influence visual cortical firing sequences such that post-experience firing was more similar to training-induced firing than pre-experience firing [29,48] (figure 2). In one study these sequences were not only replayed offline but could be induced by delivery of the stimulus that usually started the extended visual experience—a starter stimulus kicking off a full replay event [29]. In a second study [48], it was found that these sequences may have been coordinated across both visual cortex and hippocampus (figure 3). Notably, the numbers of these cortico-hippocampal co-activation events were very few (less than 20) but were multiple-fold greater than in shuffled data models. An interesting recent finding actually demonstrates much larger spatial scale repetition of wake sequences in sleep, showing travelling waves of activity across the cortical circuit [49].

In some of these cases, previously present sequences were rigorously examined and in others not. In those where they were examined, innate sequences were detected and so it may be that neocortical replay is based on refining or inducing small alterations into pre-existing sequences similar to recent findings in hippocampus [50]. The presence of these persistent underlying ‘backbone sequences’ that can be changed but remain partially present brings up the question of whether somehow the pre-existing sequences are ignored by the system and only differences make an impact on consolidation. Perhaps this occurs by a mechanism whereby the ongoing baseline sequence is always used and establishes a foundational network status (synaptic connectivity, excitability of individual elements) and only deviations from that baseline sequence drive change away from that underlying state—thereby inducing change.

## 9. Origin of sequences to be replayed

One open question is the extent to which externally imposed firing sequences during the wake state are necessary for re-sculpting offline replayed sequences. For example, in hippocampal place cell studies, animals run through many place fields in sequence and place cells are thereby forced to fire in sequence by the sequential nature of activation of their receptive fields [51]. The experience-based sequential firing of these neurons is driven by external experience and not by internal mechanisms. Are such ‘online’ externally driven activations necessary to provide substrate sequences for subsequent replay? Similar stimulus-driven sequences are clearly found in Xu *et al.* [29], Euston *et al.* [46] and probably in the navigation task used by Ji *et al.* [48], but interestingly not in Bermudez *et al.* [47] where a relatively simple 20 Hz somatosensory stimulus is delivered to the hindlimb and yet replay is still sculpted. Of course, sequential activations may have been driven by this stimulus, but the degree of sequential imposition is much smaller and certainly at a different timescale (20 Hz) from running behaviour-based sequences (multiple seconds). Additionally, certainly, cortical networks are capable of innate sequencing of cellular firing, as demonstrated by the slice work described above, and in addition cortical networks may also display novel sequences when novel non-sequenced inputs arrive. So, it may be that any new sequence, whether it originates internally or externally, is a candidate for replay. This universal plasticity principle in neocortical networks may be consistent with other neocortical plasticity paradigms such as visual cortical plasticity after simple drifting gratings are presented to passive adult animals—which imply the capacity to adapt firing rates to nearly any region-relevant stimulus regardless of salience or origin [52].

## 10. Coordination with hippocampus

Much work has examined how the hippocampus and cortex may coordinate to enable replay and memory consolidation more broadly. It is known that SWRs begin in the hippocampus [53] and modulate much of the brain [54], including the cortex [55] and thalamo–cortical spindle oscillations [55–59]. Furthermore, hippocampal SWRs are concomitant with hippocampal replay events. So, given the local hippocampal replay initiated by SWRs and their pan-brain engagement, it is possible that they essentially direct other brain regions to replay as well, including the neocortex.

Unfortunately, direct evidence regarding this hypothesis has been somewhat difficult to come by. Data to date is, however, at least consistent with synchronous replay between the two structures. Ji & Wilson [48] showed that visual cortical replay co-occurred with hippocampal replay to a degree exceeding that expected by either simple chance or data reshuffling (though still relatively rare). Jadhav *et al.* [42] also found evidence for at least some degree of co-replay between cortex (mPFC) and hippocampus. Interestingly, however, much replay in the visual cortical recordings occurred out of coordination with SWRs at the hippocampal recording site. The lack of observed coordination could be owing to under-sampling of the hippocampal SWR events which occur along the full length of the hippocampus [60] or under-sampling of neurons comprising the functional assemblies at each site. It may also be that the cortex can replay sequences with or without hippocampal inputs [61].

Further explorations into the hippocampo–cortical interaction have been numerous, but two points of particular interest have emerged—especially when looking at reactivation rather than replay studies. First, only certain specific subsets of neocortical neurons appear to be modulated by SWR activity and those co-modulated neurons, at least in the mPFC, show common coding properties to each other [42]. Second, the interaction between cortex and hippocampus is bidirectional with evidence that not only are delta waves, SWRs and spindle events temporally coupled [56,57], but spiking in the cortex predicts subsequent spiking patterns in hippocampus which then predicts further downstream spiking in the cortex [62].

In a recent study, UP state-spindle complexes were experimentally forced to occur in mPFC immediately after SWRs in hippocampus to test the import of this hippocampo–cortical coordination for memory consolidation during sleep following a spatial memory task [57]. Sleep sessions with this enforced coupling were followed by improved memory-based behavioural performance, implying that coupled activations across structures may improve overall memory consolidation.

## 11. Causal experiments of neocortical reactivation and replay

In the hippocampus, a number of causal experiments have explored the role of SWRs in effective memory consolidation. Electrical stimulation interrupting SWR events showed reduced performance and learning in post-stimulation periods relative to control animals [18,19]. More recent optogenetic experiments have shown that closed-loop prolongation of ripples leads to improved memories [20]. Finer-scale cell-specific optogenetic manipulations yielded mixed results, but generally support the notion that SWR-based replay plays a role in balancing network activity restructuring, stability and homeostasis [63–65].

In the neocortex, few studies have used optogenetics, electrical stimulation or chemogenetics to assess mechanisms and role of replay. As described above, Maingret *et al.* [57] used electrical stimulation to force UP state-spindle complexes to follow SWRs and in addition to their behavioural findings they also showed that a subset of neurons shifted the timing of their firing relative to the population activity, and became more responsive to a salient object, both phenomena not seen during control conditions. In another study, after motor learning, optogenetic inhibition of spiking during sleep UP states—states during which reactivations were observed—was correlated with reduced behavioural performance relative to control [66]. At a single cell level, without optogenetic interference in these experiments most neurons downscaled, with only stimulus-related neurons remaining up-scaled. Optogenetic inhibition during UP states led the non-specific neurons to not be downscaled either—perhaps reducing the signal-to-noise ratio of the spiking system. Similar optogenetic inhibition during nonREM sleep also prevented re-tuning of visual cortical circuits during paradigms inducing plasticity in control animals [52].

While we have begun to explore in detail the role of replay in the neocortex, further work is certainly needed in the future—perhaps with cell- and time-specific inhibition experiments to demonstrate the role of replay itself in re-sculpting neocortical networks.

## 12. Sequences existing during pre-learning states

**Figure 4.**
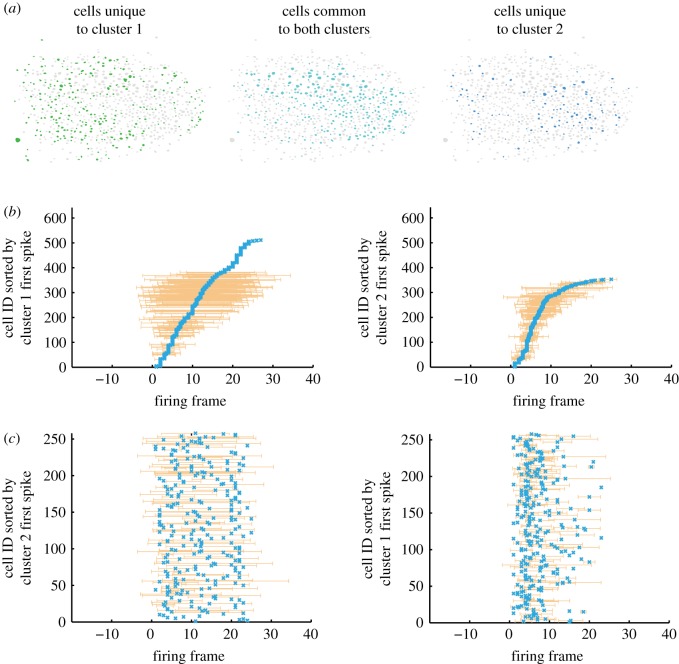
Different sequences may share the same participating neurons. (*a*) A single population of V1 neurons form different clusters in response to different circuit events, with overlapping neurons (middle). (*b*) The overlapping neurons are sorted by the averaged first firing time within each cluster, respectively. (*c*) Among shared neurons, sequences are not shared across event classes. The overlapping neurons are sorted by the averaged first firing time from another cluster. When sorted this way, sequences seen in (*b*) vanish indicating divergent circuit mechanisms dictating sequences among shared neurons. Adapted from [27]. (Online version in colour.)

As discussed above, neocortical networks demonstrate sequences regardless of recent inputs—it is unclear what these baseline or pre-learning experiences may do or offer. Naturally, sequences denoted by experimenters as ‘pre-learning’ will have actually occurred after some other learning, just not the learning imposed by the experimenter. So, one possibility is that the sequences present in these networks are always representative of prior experience-based sculpting. On the other hand, the sequences observed in slices and *in vivo* both anaesthetized and unanaesthetized may represent some baseline state of the network—which is modifiable, but towards which the network may retreat once further learning is absent. In hippocampal networks, the notion of ‘preplay’ has been explored and while certain correlated network firing sequences certainly exist, their role in hippocampus also remains unclear [5,6,14]. The same concept may apply in neocortex: one study found that multiple distinct functionally defined microcircuits in V1 cortical slices may share the same neurons but form different sequences (figure 4) [27]. This could be similar to hippocampal generation of internal modes to store novel experiences [67]. Thus, the baseline pre-experience sequences described here are approximated by the ‘backbone’ sequence concept described above—i.e. those observed when averaging across UP state sequences overall.

**Figure 5.**
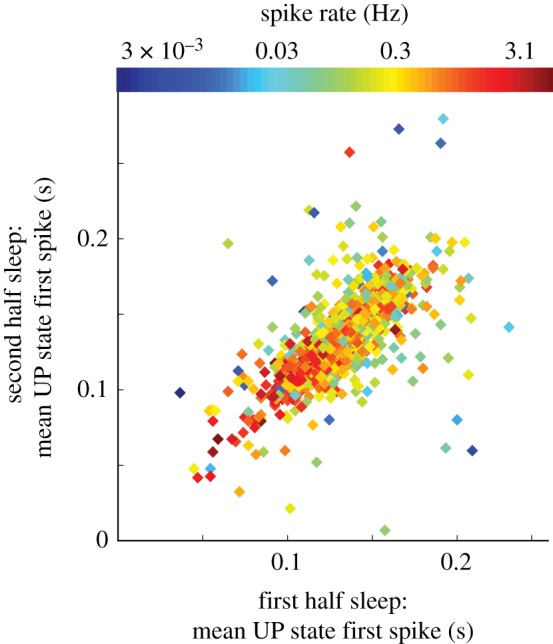
High correlation between UP state spike timing and cell firing rate. Spikes were recorded in the deep layers of frontal cortical areas in rats during daytime hours. Mean latencies to the first spike in the UP states of each cell are compared between first half and second half of sleep to show consistency of timing by each cell over sleep. Firing rates of the cells are denoted by varying colours. Note that cells with higher firing rate (redder) generally have lower first spike latencies. Adapted from [37]. (Online version in colour.)

These backbone sequences may actually correlate with basic features of the cell rather than the oft-assumed complex sequential synaptic inputs [68]. Examples of such cellular features are firing rate and excitability which themselves may relate to factors such as membrane potential, input resistance and rheobase. Specifically, two studies found that the initial spike time for any given neuron in UP state sequences was strongly predicted by the baseline spike rate of that neuron—with higher firing rate cells firing earlier in the average UP state [37,69]. This possibly implies that general excitability is a shared driving force in both firing rate and UP state spike timing (figure 5).

## 13. Sequence repetition: possible role in homeostasis

The backbone firing sequence displayed during UP states in nonREM sleep may serve a non-mnemonic purpose—it has been hypothesized to aid homeostasis. First, sleep is generally considered to serve both homeostatic [37,70–72] purposes and mnemonic purposes [39,73,74]. Much of the theory behind replay focuses on memory consolidation but the innate sequences repeated, when combined with STDP learning rules may actually induce forms of homeostasis observed in the neocortex. Second, as mentioned above, the backbone sequence tends to progress from high firing rate neurons having earlier spike timing in each UP state to low firing rate neurons having later spike timing. Third, recent work has shown that over the course of sleep, high firing rate neurons drop their firing rates while low firing rate neurons do not, or may even increase their firing rates—all in home cage sleep situations with no apparent or obvious learning to consolidate [37,75,76]—implying these spike rate changes may be homeostatic.

**Figure 6.**
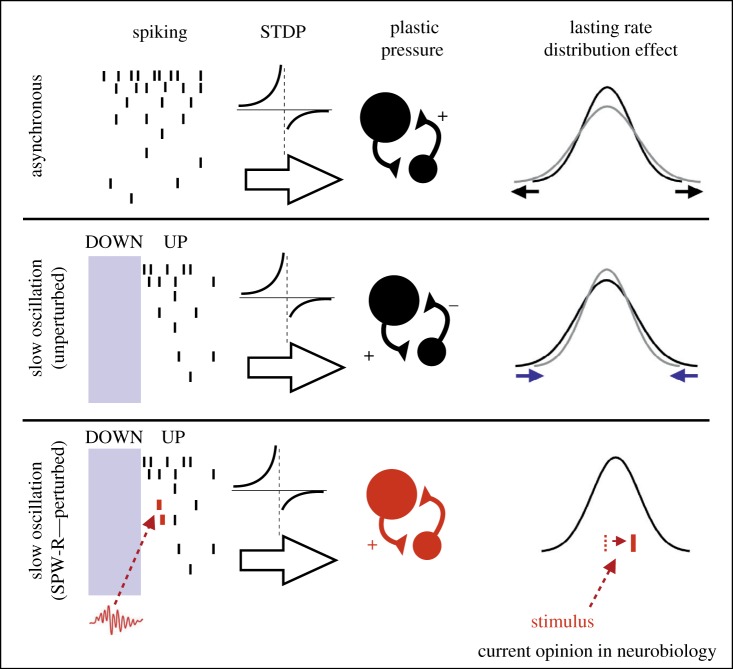
Synaptic reorganization and homeostasis based on combined spike timing and timing-based synaptic learning rules. (Top panel) During the asynchronous state (wake-like), neurons fire without gross-scale sequences. With assumed approximation, spontaneous plastic pressure owing to STDP strengthens synapses to high firing rate units, which may widen the firing rate distribution in the population (right). (Middle panel) In the synchronized state of nonREM sleep, DOWN-to-UP transitions involve spiking sequences in which high firing rate cells have earlier first spike time than low firing rate cells (left). Based on STDP learning rules this is theorized to strengthen the synapse from high firing rate neurons to low firing rate units and oppositely weakening drive onto high firing rate cells from low firing rate cells (centre). This would tighten the firing rate distribution (right). (Bottom panel) During sharp wave-ripple (SWR) events, selective memory-specific synapses may be strengthened based on the capacity of SWRs to induce firing at times not dictated by intrinsic cortical sequences. This then may promote fine-tuning of the sequence and memory formation. Adapted from [77]. (Online version in colour.)

A specific hypothesis relating this homeostasis to possible STDP mechanisms in the context of UP state sequences was recently published, stating that UP state activation sequences specifically lead to differential homeostatic pressures on high and low firing rate neurons (figure 6) [77]. The fact that high firing rate neurons may have earlier mean first spike times than low firing rate neurons may combine with STDP to render increased drive (via high rate neuron to low rate neuron potentiation) onto low firing rate neurons and decreased drive onto high firing rate neurons (via low firing rate neuron to high firing rate neuron depression) [77]. This action would specifically enable differential homeostatic actions upon neurons of different firing rates, as has actually been shown in the cortex [37,76].

The previously published notion of ‘packets’ [38] relates to the backbone sequence idea proposed there to play a homeostatic role, but the two conceptualizations are not identical. Specifically, the packet idea picks up on the notion that the high firing rate cells are reliable and may not inform as to specific behaviourally relevant data but rather encode ‘header information’ to connote basic firing, while low firing rate cells encode fine-grained behaviourally relevant information. This is important and may be correct and is consistent with the ideas stated above. However, our ‘backbone’ conception includes the fact that backbone sequences cascade from high firing rate neurons early in the sequence to low firing rate neurons later in the sequence. This important concept enables the inclusion of homeostasis based on STDP acting on this rate-related sequence.

In the hippocampus, an STDP-based decoupling was shown to weaken synaptic strength and desynchronize activity during SWR bursts both theoretically and experimentally [78]. Specifically, during bursts, the coincident spiking of connected neurons may weaken the synaptic connections between them because of the conduction delays that fall into the STDP depression time frame [78]. Interestingly, the overall firing rate distribution was found to shift toward higher rates, and is narrower for pyramidal cells in the hippocampus during nonREM [76]. Furthermore, causal sequences during SWR could interfere with the STDP-based decoupling, but these sequences may only take up a small fraction of all the bursts during nonREM [78]. These forces might counterbalance each other and prevent winner-take-all scenarios while retaining important memories [78]. Whether the cortex has analogous balance during DOWN-to-UP transition and spindles remains to be explored.

In addition to this STDP and sequence-based plasticity, neocortical UP states appear to have specific roles in inducing plasticity consistent with homeostasis: namely they downscale subthreshold synapses [79,80]. On the other hand, they may be particularly powerful at allowing potentiation of synapses leading to suprathreshold activity in postsynaptic neurons [81]. Relatedly, some UP states are accompanied by spindle oscillations during which intracellular calcium, a major plasticity signal, has been seen to be elevated [82,83] —further potentiating the role for UP states as agents of plasticity.

## 14. Conclusions and sequenced activity in the neocortex

There is evidence for repeated activity patterns in the neocortex in the forms of replay, sequences, reactivation. These three concepts, however, do not capture the fact that there is a general backbone sequence of firing in cortical networks which is able to be modified by inputs and experience in specific ways without wholly disappearing. This backbone sequence is seen in awake sensory receptive states, natural sleep as well as unnatural experimental states such as in slices and anaesthesia. It remains unclear how the backbone sequence interacts with the malleable elements of sequential activity but similar trends may be emerging in hippocampal SWR events as well [50]. One possibility is that the backbone sequence uses STDP to sculpt synaptic systems to a background state and that deviations from that sequence are used to encode synaptic or cellular states to store that new information [66].

A major feature of cortical networks, with their massively recurrent and redundant connectivity structure combined with learning rules and STDP may be the capacity to be moulded by activity be it intrinsic or extrinsic. It may be that cortical microcircuits are essentially learning machines able to learn any new firing pattern, as long as that pattern is imposed upon them repeatedly. Perhaps this capacity to learn novel information is a very fundamental adaptive purpose subserved by the cortex. This plasticity must, however, be counterbalanced by a need for static gross functionality—the capacity to consistently perceive, decide and execute based on networks. Therefore, a key element of neocortical patterned activity may be its capacity to be properly moulded while not deviating overly strongly from necessary core functionality—similar to the finding of a backbone with some super-imposed mouldability. A major task in the future will be to determine how salient information that should be adapted to is ‘tagged’ or otherwise distinguished from irrelevant information that will not re-mould the network. At the heart of the patterned activity is the capacity of the cortex to somehow properly self-manage.

## Data accessibility

This article has no additional data.
